# Isolation of Biosurfactant Producing Bacteria From Poultry Breast Skin

**Published:** 2012-08-25

**Authors:** Azizollah Ebrahimi, Najmeh Tashi

**Affiliations:** 1 Department of Pathobiology, School of Veterinary Science, Shahrekord University, Shahrekord, IR Iran

**Keywords:** Biosurfactant, Chicken, Turkey, Quail, Iran

## Abstract

**Background:**

Biosurfactants are surface-active compounds produced by some microorganisms.

**Objectives:**

In this study, we collected surface skin samples from breast of poultry (chicken, turkey, and, quail) and screened for biosurfactant-producing bacteria. We also determined the genera of cultured strains.

**Materials and Methods:**

33 hemolytic bacterial strains (15, 11, and 7 isolates from chicken, turkey, and quail, respectively) were isolated; oil spreading (OS) and bioemulsifying activities were measured for all isolates.

**Results:**

Two isolates of chicken (6.06%), three of turkey (9.1%), and three of quail (9.1%) were positive in all examinations (hemolysis, emulsification index (E24) and oil spreading). In total, eight isolates (24.24%) were positive in all examinations, out of them, seven isolates (87.5%) were gram positives, mainly belonged to Bacillus spp., Staphylococcus spp. and Lactobacillus spp. 31 isolates (93.9%) (out of 33 hemolytic isolates) were positive in oil spreading test while only eight isolates (24.24%) were positive in E24 test.

**Conclusions:**

The results showed that biosurfactant-producing bacteria are distributed in breast skin surface of examined birds. Further investigation about the composition of biosurfactants and phylogenetic determination of biosurfactant producing bacteria is suggested.

## 1. Background

Biosurfactants are a unique class of compounds that have been shown to have a variety of potential applications in remediation of organic-and metal-contaminated sites, in the enhanced transport of bacteria, in enhanced oil recovery, as cosmetic additives, and in biological control (1). They are amphiphilic compounds produced on living surfaces, mostly on microbial cell surfaces, or excreted in extra cellular spaces and contain hydrophobic and hydrophilic moieties that confer the ability to accumulate between fluid phases, thus reducing surface and interfacial tensions (2). Rosenberg and Ron (3) suggested that biosurfactants can be divided into low-molecular-mass molecules, which efficiently lower surface and interfacial tensions, and high- molecular- mass polymers, which are more effective as emulsion-stabilizing agents. Recently, several groups have presented fascinating data suggesting that biosurfactants are important agents for microbial growth and survival in the environment. For example, surfactin production is necessary for fruiting body formation by Bacillus subtilis (4). Apart from their obvious role as agents that decrease surface and interfacial tensions leading to promote the formation and stabilization of emulsions, surfactants may exhibit several other functions. They improve consistency and texture of fat-based products (5). Several biosurfactants have shown antimicrobial action against bacteria, fungi, algae, and viruses (6). There are many advantages of biosurfactants compared to their chemically synthesized counterparts. Research in this subject will make biosurfactants as highly sought-after biomolecules in terms of their present and future applications such as fine specialty chemicals, biological control agents, and new generation of molecules for pharmaceutical, cosmetic, and health care industries. The presence of biosurfactant producing bacteria greatly depends on the composition of environment in which they reside. Uropygial gland of birds produces lipids and waxy sebum that coat the bill and are transferred to the plumage during preening. Bandyopadhyay and Bhattacharyya (7) found that domestic fowl uropygial secretions enhanced the growth of the bacteria Staphylococcus epidermidis, Streptomyces spp. and Proteus spp., but inhibited the bacterium Bacillus anthracis. Thus, the effects of uropygial oil on microbial communities of bird’s skin appear to be complex.

## 2. Objectives

There are no reports in terms of isolation of biosurfactant producing bacteria from birds or animals. The aim of the present study was to investigate biosurfactant producing bacteria (BPB) habitats on breast skin (as oily skin area) of female and male chicken, turkey, and quail.

## 3. Materials and Methods

### 3.1. Sample Collection

The study was carried out between April to September 2011 on 60 female and male chickens, turkeies and quails (each species 20, each sex 10). They were randomly selected from poultry farms in Shahrekord district in west center of Iran. All animals were adults and were found to be apparently healthy. Samples were collected by rubbing sterile cotton-tipped applicator sticks on skin of breast areas. The surfaces were thoroughly rubbed by rolling the wet swabs to attain effective contact. The swabs were put in separate sterile test tubes containing Stuart transport media (Quelab cat. QB-65-5015) labeled and kept in a cool box and transported to the Veterinary Microbiology Laboratory of Veterinary College of Shahrekord University for further processes. For bacteriological examination, the swabs were removed from the tubes and streaked over the plates of blood agar (Scharlau 01-352, EU), supplemented with 7% sheep blood. The streaking was further spread with inoculating loop to aid colony isolation. The plates were labeled and incubated aerobically at 37°C for 24-48 hours (h) (8). One colony was selected from those colonies that already showed similar morphologies and sub-cultured on blood agar plates for further analysis.

### 3.2. Screening Methods

#### 3.2.1. Hemolysis Test

The first screening test for identification and isolation of BPB is hemolysis test (9). Each strain was streaked on blood agar medium and incubated for 48 h at 37°C to assay hemolytic activity. The plates were visually inspected for zones of clearing around the colonies, indicative of biosurfactant production.

#### 3.2.2. Identification of BPB

One colony was selected from those hemolytic colonies that have similar morphologies and sub-cultured on blood agar plates for further analysis. After gram staining, catalase and oxidase tests, identification of isolated microorganisms was performed using a standard biochemical scheme according to Balows et al. (10). Hemolytic isolates were inoculated into tubes containing Luria Bertani broth (LB, Biomark- B699) media and incubated at 37°C for 72 h with shaking (~ 50 rpm). One tube of sterile LB media was considered as control for each set of cultures.

#### 3.2.3. Oil Spreading Test

To apply oil spreading technique (OS), 50 ml of distilled water was added to a large petri dish (25 cm diameter) followed by addition of 20 µl of n-Decane (Merck, UN 2247) to the surface of the water. 10 microliters of cell-free broth of LB culture (Centrifuged at 10000 rpm for 10 min.) were then added to the surface of oil (11). Diameter of clear zone on the oil surface was measured. The diameters of triplicate samples from the same culture of each strain were determined.

#### 3.2.4. Emulsification Test (E24)

Emulsifying capacity was evaluated by an emulsification index (E24). This index of culture samples was determined by adding 1.5 ml of kerosene and 1.5 ml of cell-free broth in test tube, vortexed at high speed for 2 min and allowed to stand for 24h and 72h. The E24 (and E72) index is given as percentage of the height of emulsified layer divided by the total height of the liquid column (cm). The percentage of emulsification index calculated by using the following equation (12), E24 = Height of emulsion formed x 100/total height of solution. Centrifuged samples of incubated tubes of sterile LB were used as control for each test strain.

### 3.3. Data Analysis

Chi square test was used to compare differences in numbers of hemolytic, OS, and emulsification positive isolates between three poultry species and two sexes of each species.

## 4. Results

After culture and incubation of 60 samples (20 from each animal species, 10 females and 10 males) 33 hemolytic strains (15, 11, and 7 strains from chicken, turkey and quail, respectively) were isolated. Differences of numbers in three poultry species were not statistically significant, (P > 0.05). OS and bioemulsifying activities were measured for all isolates (Table). Two isolates of chicken (6.06%), three of turkey (9.1%), and three of quail (9.1%) were positive in all examinations (hemolysis, E24, and OS). Differences of above numbers in three poultry species were not statistically significant, (P > 0.05). In total, eight isolates (24.24%) were positive in all examinations out of them seven isolates (87.5%) were gram positives mainly belonged to Bacillus spp., Staphylococcus spp. and Lactobacillus spp. 31 isolates (93.9%) (out of 33 hemolytic isolates) were positive in oil spreading test, while only eight isolates (24.24%) were positive in E24 test (Table).

**Table:** Bacteria Isolated From Poultry, Turkey and Quail

		Female			Male		
		E24 a, %	E72 a, %	OS a, SD a, cm	E24, %	E72, %	OS, SD, cm
Chicken	*Staphylococcus spp./Staphylococcus spp*	32	40	5.75 ± 0.1	40.2	40	6 ± 0.1
	*Lactobacillus spp/Aeromonas spp*	36	40	4.35 ± 0.3	52	44	4.5 ± 0.05
	*Staphylococcus spp./Bacillus spp.*	32	36	5.3 ± 0.1	40	32	5.3 ± 0.1
	*Staphylococcus spp./Lactobacillus spp.*	28	36	3.2 ± 0.0	28	20	6 ± 0.1
	*Bacillus spp./Acinetobacter spp.*	40	36	5.5 ± 0.1	48	40	6 ± 0.2
	*Acinetobacter spp./Staphylococcus spp.*	40	28	4.2 ± 0.1	52	32	3.05 ± 0.2
	*-/Bacillus spp.*	-	-	-	44	40	5.7 ± 0.1
	*-/Staphylococcus spp.*	-	-	-	36	40	3.1 ± 0.2
	*-/Bacillus spp.*	-	-	-	40	40	6.3 ± 0.1
	*Control*	50	50	1.75 ± 0.3	50	50	1.75 ± 0.1
Turkey	*Staphylococcus spp./Staphylococcus spp.*	40	48	3.8 ± 0.1	37	44	4.4 ± 0.1
	*Lactobacillus spp./Staphylococcus spp.*	48	56	3.45 ± 0.05	44	48	6.6 ± 0.2
	*Bacillus spp./Staphylococcus spp.*	36	40	2.5 ± 0.4	33	40	3 ± 0.14
	*Bacillus spp./Bacillus spp.*	48	44	6.6 ± 0.24	37	36	4.8 ± 0.24
	*Saphylococcus spp./Streptococcus spp*	44	44	2 ± 0.1	40.7	32	6.4 ± 0.1
	*Control*	44	44	2.05 ± 0.34	40.7	44	2.05 ± 0.34
Quail	*Bacillus spp./Staphylococcus spp.*	45	55	5.5 ± 0.05	55	65	3.3 ± 0.05
	*Bacillus spp./Staphylococcus spp.*	45	50	5.6 ± 0.24	40	40	4.1 ± 0.1
	*Lactobacillus spp./Staphylococcus spp.*	60	60	5.4 ± 0.1	65	70	4.9 ± 0.05
	*Staphylococcus spp/-.*	40	50	3.6 ± 0.44	-	-	-
	*Control*	50	50	2.5 ± 0.05	50	50	2.5 ± 0.05

^a^Abbreviations: OS, Oil spreading; SD, Standard deviation; E24, Emulsification Index (24 hours); E72, Emulsification Index (72 hours)

## 5. Discussion

Hemolytic activity appears to be a good screening criterion in the search for BPB (8). Such screening can be used to limit the number of samples. Further screening of BPB is generally carried out using monitoring parameters that estimate surface activity, such as OS test and the ability to emulsify oils (13). Using OS test, comparatively high abundances of surfactant-producing bacteria (31 (93.9%) out of 33 hemolytic isolates) were isolated from the examined birds. This technique is reliable in biosurfactant detection as determined by surface tension measurement (13). However, some skin areas that were not studied here may contain even more surfactants produced by BPB compared to studied areas. We could not find reports in respect to BPB isolation from birds or animals; however our other works indicated that BPB are also present on oily areas of small animal and ruminant skin (14, 15). The presence of BPB has been also described in the guts of some marine invertebrates (16). A relatively biosurfactant producing Bacillus spp., Staphylococcus spp., and Lactobacillus spp. domination were represented in isolated strains. This distribution may represent the ability of microorganisms to survive in breast skin areas of examined birds. In all female poultry, the highest OS values were mostly exhibited by Bacillus spp., but in males it was showed by Staphylococcus spp. isolates. On other hand, in females of turkey and quail, highest values of emulsification tests were shown by Lactobacillus spp., but in males of these two species highest values were shown by Staphylococcus spp. isolates, (Table).

Comparing the numbers of positive BPB isolates, significant differences were not observed between two sexes of examined poultries, (P > 0.05). The biosurfactant activity in Bacillus spp. isolated from diesel oil was documented by Singh and Lin (17). Tabatabaee et al. (18) also supports the biosurfactant activity of this bacteria. Rodrigues et al. (19) demonstrated biosurfactant activity of Lactobacillus spp. and that cheese whey can be used as an alternative medium for biosurfactant production by this bacteria. Production of biosurfactants by Staphylococcus spp. is also documented (20). Biosurfactant production by many of the isolated strains suggests that the resident bacteria could be a source of surfactants in the studied skin areas. Function and composition of surfactants in the organisms of examined areas have not been established. It might be suggested that the surfactants assist in surface fat layer removal process by solubilizing hydrophobic fat layer or preventing destructive function of skin lytic substances. It may also dissolve organic matter of skin surface secreted by different body systems or play some roles in bacterial community formation of skin surfaces. From a clinical perspective, at least one biosurfactant, rhamnolipid produced by Pseudomonas aeruginosa, has a role in pathogenesis of this opportunistic pathogen (21). Biosurfactants are often superior to commercial surfactants at solubilizing different chemicals and are more easily biodegraded (6). Genera of biosurfactant producing bacteria isolated from the studied areas shown in Table, are well documented to be present in different oily environments such as potato process effluents, cassava flour waste water, and oil reservoirs for Bacillus spp. (6, 18). The results indicated that biosurfactant-producing bacteria were distributed on poultry breast skin. Microorganisms isolated in this study could be valuable sources for novel biosurfactants. Further investigation about the composition of biosurfactants and phylogenetic determination of biosurfactant producing bacteria is suggested.
